# Environmental Control of Phase Transition and Polyp Survival of a Massive-Outbreaker Jellyfish

**DOI:** 10.1371/journal.pone.0013793

**Published:** 2010-11-03

**Authors:** Laura Prieto, Diana Astorga, Gabriel Navarro, Javier Ruiz

**Affiliations:** Department of Ecology and Coastal Management, Instituto de Ciencias Marinas de Andalucía (CSIC), Cádiz, Spain; University of Canterbury, New Zealand

## Abstract

A number of causes have been proposed to account for the occurrence of gelatinous zooplankton (both jellyfish and ctenophore) blooms. Jellyfish species have a complex life history involving a benthic asexual phase (polyp) and a pelagic sexual phase (medusa). Strong environmental control of jellyfish life cycles is suspected, but not fully understood. This study presents a comprehensive analysis on the physicochemical conditions that control the survival and phase transition of *Cotylorhiza tuberculata*; a scyphozoan that generates large outbreaks in the Mediterranean Sea. Laboratory experiments indicated that the influence of temperature on strobilation and polyp survival was the critical factor controlling the capacity of this species to proliferate. Early life stages were less sensitive to other factors such as salinity variations or the competitive advantage provided by zooxanthellae in a context of coastal eutrophication. Coherently with laboratory results, the presence/absence of outbreaks of this jellyfish in a particular year seems to be driven by temperature. This is the first time the environmental forcing of the mechanism driving the life cycle of a jellyfish has been disentangled via laboratory experimentation. Projecting this understanding to a field population under climatological variability results in a pattern coherent with *in situ* records.

## Introduction

The wide-ranging environmental and economic impacts of large accumulations of gelatinous zooplankton have resulted in extensive recent scientific interest [1]–[7]. A number of causes have been proposed to account for the occurrence of gelatinous zooplankton (both jellyfish and ctenophore) blooms, such as eutrophication [8], increases in artificial hard substrata for attachment of polyps [9], [10], exotic translocations [11], over-fishing [12] and climate change [13]. However, no consensus has been reached, as proposed causes vary depending on the focus of the study, i.e. global or local [14].

Many jellyfish species have a relatively complex life history involving a benthic asexual stage (polyp) and a pelagic sexual stage (medusa). This makes it complicated to determine which phase of the life cycle is the most affected by varying environmental conditions, and potentially controls the presence/absence of jellyfish outbreaks. Polyps (scyphistoma) form when planulae settle on a benthic surface. Planulae are ciliated planktonic larvae that result from sexual reproduction of adult medusae. Polyps reproduce asexually by budding, which eventually can produce colonies of millions of individuals. The transition of jellyfish from the benthic to pelagic stages occurs during strobilation. Through this process, individual polyps metamorphose to form the juvenile pelagic stage (free-swimming ephyra) that mature into adult medusae. In some species, strobilation is apparently triggered by the seasonal warming of the water [15]–[17]. Depending on the survival rates of ephyrae, successful strobilation of great numbers of polyps can generate large jellyfish blooms. Although of less societal impact, the benthic phase is crucial for the population dynamics of jellyfish, because polyps usually persist for longer than the medusae. Though less studied than the pelagic stage, factors that affect the growth and survival of polyp colonies are critical for determining the subsequent abundances of medusae [18]–[22].

Currently there is concern that jellyfish are becoming more prevalent in a variety of regions around the Mediterranean Sea [9], [23]–[25]. *Cotylorhiza tuberculata*, an exotic scyphozoan in the Mar Menor (western Mediterranean), was selected as a case study for parameterization of an invasive jellyfish species. The Mar Menor is the largest coastal lagoon in the western Mediterranean with a surface area of about 135 km^2^ and a mean depth of about 3.5 m. The lagoon is exposed to intensive tourism. There is no mention of *C. tuberculata* in the lagoon before its connection with the Mediterranean was made deeper and wider in the 1970s to facilitate navigation. Since the early 1990s the high abundance of *C. tuberculata* during summer has become an increasing problem for the recreational use of the lagoon. Local authorities have implemented programs for the removal of adults by means of fishing vessels or the use of nets to protect bathing areas from medusae. Previously these programs have removed over five thousands tons of *C. tuberculata* during the most abundant summers [26].

To understand the mechanisms driving *C. tuberculata* blooms in the hypersaline Mar Menor coastal lagoon, several experiments were performed to parameterize the physical control of phase transition and polyp survival. Because of its shallow and enclosed nature, the physical environment of the Mar Menor lagoon is more extreme than the open Mediterranean Sea. Thus, we hypothesise that the resistance of *C. tuberculata* early life stages to the range of environmental conditions in the lagoon is the origin of the inter-annual presence/absence of *C. tuberculata* outbreaks. The experiments were performed on successive stages of the life cycle and the results obtained clearly identify the source of inter-annual fluctuations in *C. tuberculata* populations.

## Materials and Methods

The Instituto de Ciencias Marinas de Andalucia (ICMAN) of the Consejo Superior de Investigaciones Cientificas (CSIC) approved this study.

**Figure 1:**
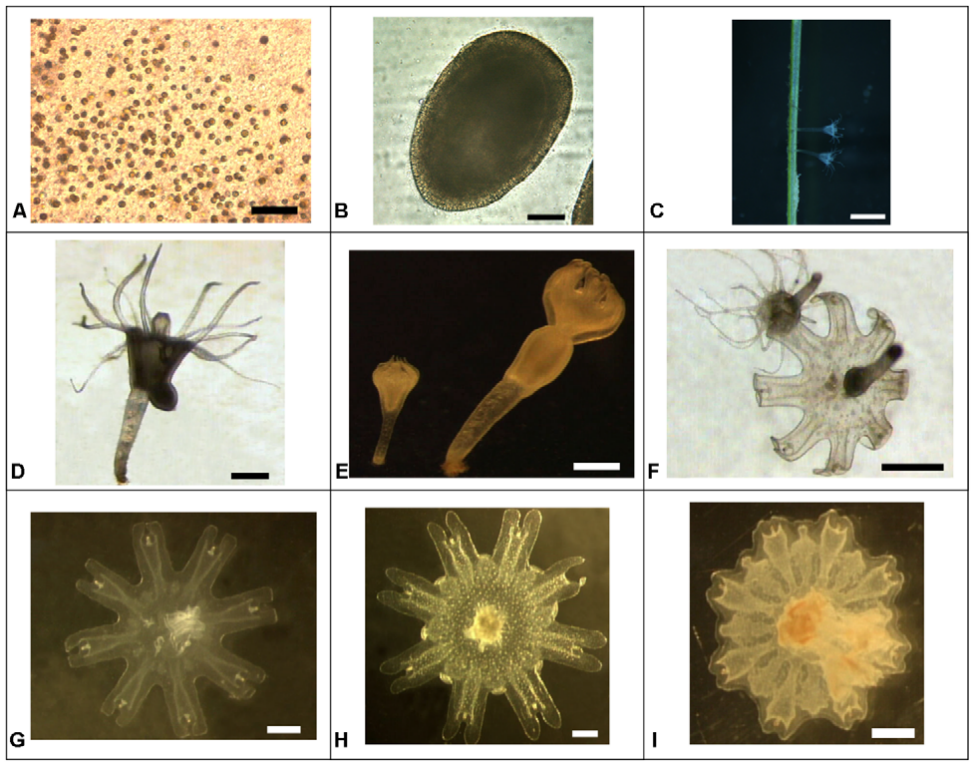
*Cotylorhiza tuberculata*. Various stages of its life history. Pictures obtained during the simulation of *Cotylorhiza tuberculata* life cycle in the laboratory. **A**) Symbiotic zooxanthellae in the tissue of the mature medusae (scale 50 μm); **B**) Planula (scale 50 µm); **C**) Detail of scyphistomae on the leaves of the marine angiosperm *Cymodocea nodosa* (scale 2 mm); **D**) Budding scyphistoma (scale 500 μm); **E**) Scyphistoma initiating strobilation (scale 500 μm); **F**) Scyphistoma totally developed and scyphistoma about to liberate an ephyra (scale 500 μm); **G**) Ephyra (500 μm); **H**) Metaephyra (scale1 mm); **I**) Young medusa (scale 1 mm). Authors of the pictures: C. Lama: A; J.B.Ortiz: B; L.Prieto: C; D.Astorga: D–I.

The life cycle of *C. tuberculata* follows the general schedule of metagenesis of scyphozoans. This cycle was replicated in the laboratory starting with gravid females collected in late September of 2006, 2008 and October 2009. The mature jellyfish liberated a large amount of ciliated planktonic larvae (planulae) while in captivity. These planulae were allocated to different glass flasks filled with water from Mar Menor for the subsequent development of the life cycle and its parameterization in terms of vulnerability to the physical environment. The experiments were performed on successive stages of the life cycle (Table 1). Those stages were examined and photographed under a binocular microscope (Fig. 1).

**Table 1:** Summary of the different experiments performed on the life cycle of *Cotylorhiza tuberculata*.

Process of the Life-Cycle	Phase/Phase Transition	Test	Code of the Experiment [num. of treatments]	Acclimation	Description of the Treatments	Replicates per treatment [average num. of individuals per replicate]
**Planulae Settlement**	pelagic → benthic	Temperature	Exp. A[5]	None	4°C, 12°C, 20°C, 24°C and 30°C, all at 47 of salinity	3 [120]
		Salinity	Exp. B[4]	None	20, 35, 47 and 53 of salinity, all at 24°C	3 [120]
**Polyp Survival**	benthic	Salinity fluctuations	Exp. C[4]	7-10 days at given salinity	47-40-35 salinity,20-35 salinity,47-35 salinity	3 [20]3 [20]4 [20]
		Light, nutrients and food (zooplankton as prey)	Exp. D[6]	6 months at 17.5°C *	light-fed-nutrients,dark-fed-nutrients,light-unfed-nutrients,dark-unfed-nutrients,light-unfed-nonutrients,dark-unfed-nonutrients	3 [35]
		Low temperatures	Exp. E[4]	4 months at 16°C *	2°C, 4°C, 9°C and 16°C	3 [13]
**Strobilation**	benthic →pelagic	Constant temperatures	Exp. F[4]	35 days at given temp.	17.5°C, 19°C, 20°C and 21°C	1 [42]
		Incremental temperatures	Exp. G[5]	5 months at 17.5°C *	18°C, 19°C, 20°C, 21°C and 23°C	3 [50]
		Increased temp. and food availability	Exp. H[2]	7 months at 17.5°C *	fed and unfed, all at 26°C	3 [46]
		Temp. fluctuations at synoptic time scales	Exp. I[4]	35 days at 20°C	1, 3, 7, and 15 days at 16°C, then put back at 20°C (45 days)	3 [30]

*Acclimation times resulting of the period since the start of the polyp culture until the beginning of the experiment at regular maintenance in culture conditions: filtered seawater, fed once a week with rotifers, light photoperiod (12∶12).

Planulae appear in late summer and early autumn when adult females reach maturity. Consequently, planulae survival is linked to lagoon conditions in that season, when the physical environment can change abruptly, in association with the passage of low pressure weather systems across eastern Spain. Average water temperature in Mar Menor ranges from 10°C in winter to 31°C in summer whereas salinity is more stable with values between 42 and 47 [27].

### Transition from pelagic to benthic phase: planulae settlement

*C. tuberculata* planulae attach to the substrate and develop tentacles to feed on micro-zooplankton. Experiments were conducted to check planula survival and the sensitivity of this process (fixing and tentacle developing) to the range of temperature and salinities measured in the lagoon. Sub-samples of equal volume were obtained from an agitated container with planulae, resulting in a set of flasks containing approximately 120 planulae each. Glass slides were used as subtrate for the planulae to attach. Each of these flasks received a different treatment to test for sensitivity to temperature and salinity.

#### Experiment A: Settlement at constant temperatures

Five treatments with three replicates each, at 4, 12, 20, 24 and 30°C, were conducted to check for the sensitivity of phase transition to temperature. Salinity was fixed at 47 in all treatments, which was the salinity of the Mar Menor at the time the planulae were collected. Temperatures were maintained by water baths (FRIGITERM of P.Selecta). Polyps with tentacles were counted for each replicate from the beginning of the experiment, which lasted 71 days.

#### Experiment B: Settlement at constant salinities

Four treatments with three replicates each, at 20, 35, 47 and 53, were conducted to check for the sensitivity of planulae to a wide range of salinities. Temperature was fixed at 24°C in all experiments, the temperature of Mar Menor when mature *C. tuberculata* were sampled in late September 2006. The salinity of 53 was chosen to check for viability of planulae at the maximum salinity that the lagoon had before the connection to the Mediterranean Sea was enlarged. The lowest salinity (20) represented values observed near the main entrance of freshwater (El Albujón) during an extreme rainy season (which are rather infrequent).

### Benthic phase: Polyp survival

The polyp culture was maintained with filtered seawater, in an incubator (IBERCEX F-4) with light photoperiod (12∶12) and fed once a week with rotifers. After one hour feeding, the water was replaced with new seawater. The selection of rotifers was performed based on the small size of the *C. tuberculata* polyps (calyx around 0.5 mm). Light intensity was 360 µmol quanta m^−2^ s^−1^. The light source consisted of four Philips master TL-D 18W/840 fluorescence lamps. The light intensity was measured using a calibrated Biospherical Instruments (San Diego, CA) QSL2100 sensor.

#### Experiment C: Polyp survival with salinity fluctuations

Four treatments with three replicates each were conducted to analyse polyp survival through possible salinity fluctuations occurring in the lagoon during the seasonal cycle. The change of water salinity was performed after feeding the polyps. Each treatment is detailed in Table 2. In “*Treatment 1*” polyps experience a change from 47 to 40, the same polyps were later changed from 40 to 35 (“*Treatment1.2*”). In “*Treatment 2*” the polyps experience a direct change from 20 to 35 and “*Treatment 3*” the polyps experience a single change from 47 to 35.

**Table 2:** Percentage polyp survival during salinity fluctuations (Experiment C).

Treatment	Salinity 1	Salinity 2	% Survival		SD	n
1	47	40	93	±	34	3
1.2	40	35	94	±	8	3
2	20	35	68	±	37	3
3	47	35	105	±	30	4

In “*Treatment 3*” polyps exclusively experienced one change of salinity from 47 to 35, while a previous test checked first a change from 47 to 40 (“*Treatment 1*”), followed by a change from 40 to 35 “*Treatment 1,2*”. In “*Treatment 2*” polyps experienced one change of salinity from 20 to 35. *SD* is the standard deviation. *n* is the number of replicates and in each replicate were on average 20 polyps.

#### Experiment D: Polyp survival at different light, nutrient and food regimes

In contrast to previous reports for *C. tuberculata* [28], zooxanthellae were already present during the polyp phase in the individuals used in this study. It has been argued that symbionts contribute to the metabolism of *C. tuberculata* and, owing to the recent eutrophication of the lagoon, there is a direct cause-effect connection between the increased input of nutrients and the outbreaks of *C. tuberculata* in Mar Menor [29]. Considering these arguments, an experiment varying food and light was used to test their differential effects on the survival of *C. tuberculata* polyps. Three flasks (replicates) containing between 20 and 50 polyps were treated according to different combinations of light, feeding and nutrients (see Table 1). In a first combination of treatments, polyps inside all flasks were unfed, and the two fixed and orthogonal factors tested were absence/presence of light and absence/presence of nutrients in the water. In a second combination of treatments, all the flasks had nutrient-rich water, and the two factors tested were absence/presence of light and fed/unfed. When the treatment was fed, rotifers were added as food once a week. After one hour feeding, the water was replaced with new seawater. In the unfed treatment, the polyps were starved by not adding any zooplankton food during the experiment; however, the water was replaced with new seawater as in the feeding treatment. Illuminated flasks were exposed to a photoperiod (12∶12), whereas dark flasks received no light during the experiment. Light intensity was 360 µmol quanta m^−2^ s^−1^. The light source consisted of four Philips master TL-D 18W/840 fluorescence lamps, suitable for zooxanthellae growth as the wavelength was 300–700 nm [30]. The incubator was an IBERCEX F-4. To check for the potential effect of nutrient depletion on the survival of polyps, via nutrient limitation of symbionts, replicates were grown in artificial seawater without soluble nitrogen and phosphorous (treatment of absence of nutrients). Additional flasks were held as control in conditions of photoperiod, water with nutrients and feeding with rotifers. Nutrient concentrations were 0.2 mM of dissolved nitrogen (nitrite, nitrate and ammonium) and 0.7 µM of phosphorous, which are within the ranges found in the lagoon [31].

#### Experiment E: Polyp survival at low temperatures

To check the polyp's ability to survive under low winter temperatures, three replicates were maintained at 2, 4, 9 and 16°C for 32 days. Before the experiment, all the replicates were maintained in culture at 16°C for 4 months in filtered seawater, fed once a week and had a light photoperiod (12∶12). The change of temperature was performed in one step and temperatures were maintained by water baths (FRIGITERM of P.Selecta).

#### Transition from benthic to pelagic phase: strobilation

*C. tuberculata* transition from benthic to pelagic stages occurs through the strobilation process. In other species, strobilation is apparently triggered by the seasonal heating of the water [15]–[17]. Experiments were conducted to evaluate the influence of temperature and feeding conditions on this process. Also, a detailed monitoring of polyps during strobilation was conducted at a temperature of 19°C with fed polyps, by observing them under steroscope everyday.

#### Experiment F: Strobilation at constant temperatures

To check for the effect of temperature, polyps were acclimated to 17.5, 19, 20 and 21°C during more than one month (35 days). Each treatment in this experiment included 98, 28, 13 and 29 polyps, respectively. Polyps were held in artificial seawater without iodine during this period to prevent strobilation. The lack of iodine is known to inhibit strobilation of scyphozoans like *Aurelia aurita* [32]–[34] or the symbiotic *Cassiopea* spp. [35]. After adding iodine, the polyps were kept at the same temperatures of acclimation. During the experiment, polyps were fed once a week. The addition of iodine was carried out by replacing the artificial seawater without iodine by natural filtered seawater after feeding at Day 35.

#### Experiment G: Strobilation in incremental temperature increase

To test the effect of temperature change on strobilation, polyps were acclimated to 17.5°C for 5 months in filtered natural seawater and fed once a week. The temperature change treatments were 18, 19, 20, 21, 23°C. The temperature was changed in one single step and temperatures were maintained by water baths (FRIGITERM of P.Selecta). Each treatment was done in triplicate including a minimum of 50 polyps per flask. This experiment lasted for 70 days and polyps were not fed during that period. The control treatment was maintained at 17.5°C.

#### Experiment H: Strobilation with increased temperature and different feeding conditions

This experiment was performed to check the effect of feeding on strobilation. Polyps that were acclimated to 17.5°C for 7 months in filtered natural seawater and fed once a week were exposed to 26°C to invoke strobilation and one treatment continued to be fed once a week whereas the other treatment was unfed. The change of temperature was performed in one step and temperatures were maintained by water baths (FRIGITERM of P.Selecta). Each treatment was done in triplicate with between 40–52 polyps per flask (a density low enough to avoid potential competition for food). This duration of the experiment was 70 days; which was chosen so as to be comparable with the previous experiment.

#### Experiment I: Strobilation with temperature fluctuations at synoptic time scales

Experiments G and H in the previous section, demonstrated how polyps acclimated to low temperatures for long periods could be stimulated to induce strobilation by an increase in temperature. Both experiments simulated the spring heating of the water (occurring only once a year). In nature, polyps are also exposed to temperature fluctuations at lower time scales due to synoptic (meteorological) events. Four treatments with three replicates each were conducted to check the seasonal against the synoptic effect (meteorological event with a time scale between days to weeks) of the environment temperature to trigger the strobilation process. Polyps that were acclimated per 35 days to 20°C were exposed to 16°C for 1, 3, 7 or 15 days (three replicates per time lag with an average of 30 polyps each), and after that lower temperature short exposure, they were transferred again to 20°C. Then, the observation of possible strobilation was perfomed for the following 45 days. Both changes of temperature were performed in one step and temperatures were maintained by water baths (FRIGITERM of P.Selecta).

### Statistics

Statistical analyses of the data were performed using Matlab™ software. ANOVA assumptions were tested on data sets prior to evaluation of variance between treatments. If data failed normality and equality of variances and homogeneity couldn't be established using the respective transformations, non-parametric Kruskal-Wallis analysis of variance was used.

## Results

### Transition from pelagic to benthic phase: planulae settlement

#### Experiment A: Settlement at constant temperatures

**Figure 2:**
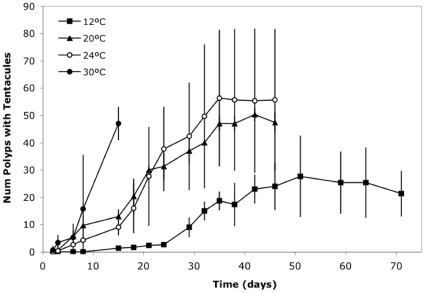
Number of planulae that fix and develop tentacles in different incubation temperatures (Experiment A). Filled Circle, empty circle, triangle and square stand for 30, 24, 20 and 12°C, respectively. An experiment of 4°C was also conducted but no polyps developed tentacles. Error bars are the standard deviations of the three replicates'.

At 4°C very few planulae attached to the substrate and none of them developed tentacles to complete metamorphosis into a fully developed polyp. This extreme temperature is never registered during autumn in Mar Menor, but was nevertheless included to check for a threshold temperature for survival. Approximately 40% of planulae in the 20, 24 and 30°C treatments settled and metamorphosed into polyps (H = 5.57, p>0.05, around 50 fully developed polyps per flask). The time taken for planulae to settle, however differed depending on temperature (H = 11, p < 0.05). At 30°C planulae settled within 15 days, but at 20°C more than 30 days were required for settlement (Fig. 2). In the case of 12°C, at Day 71 of experiment, and after 29 days of no change in the remaining planulae (between days 42–71: F = 1.19, p>0.05), only an average of 25% settled and metamorphosed into polyps (Fig. 2).

#### Experiment B: Settlement at constant salinities

**Figure 3:**
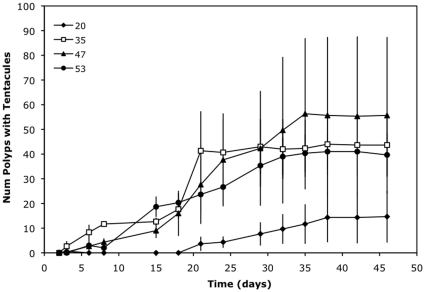
Number of planulae that fix and develop tentacles under different salinities (Experiment B). Rhomboid, empty square, triangle and circle stand for 20, 35, 47 and 53 of salinity, respectively. Error bars are the standard deviation of the three replicates'.

Except for very low values (20 of salinity) the process of polyp formation showed little sensitivity to the wide range of salinities tested (from 20 to 53) (Fig. 3: F = 1.47, p>0.05). Consequently, salinity exerts little control on the process of polyp formation and its role in the population dynamics within the Mar Menor is small if any.

### Benthic phase: Polyp survival

#### Experiment C: Polyp survival with salinity fluctuations

Polyps were not affected by salinity changes once fully developed (Table 2). The percentage of survival was close to 100% (average 91%) and in all cases, independent of whether the drop in salinity occurred in one or two steps (F = 1.47, p>0.05 see Table 2). Polyp survival was lower (68%) only in the case of a one step increase of salinity from 20 to 35.

#### Experiment D: Polyp survival at different light, nutrient and food regimes

Manifest decreases in the number of polyps only occurred for flasks with the starvation treatment but in presence of both light and nutrients, although a 3-way ANOVA showed that the different factors analyzed had no effect on the survival of polyps (F_light_ = 1.16, F_food_ = 1.62, F_nutrients_ = 2.36, p>0.05). Interactions between light and/or food or nutrients did not affect the survival (F_light*food_ = 0.39, F_light*nutrients_ = 0.22, p>0.05). Comparing the control and the no light treatment (dark-fed-nutrients), asexual reproduction by budding at a constant temperature was slightly increased by the absence of light (i.e. as the total number of polyps increased with time in the no-light treatment compared to the control), but the differences were not significant (F = 1.35, p>0.05).

#### Experiment E: Polyp survival at low temperatures

**Figure 4:**
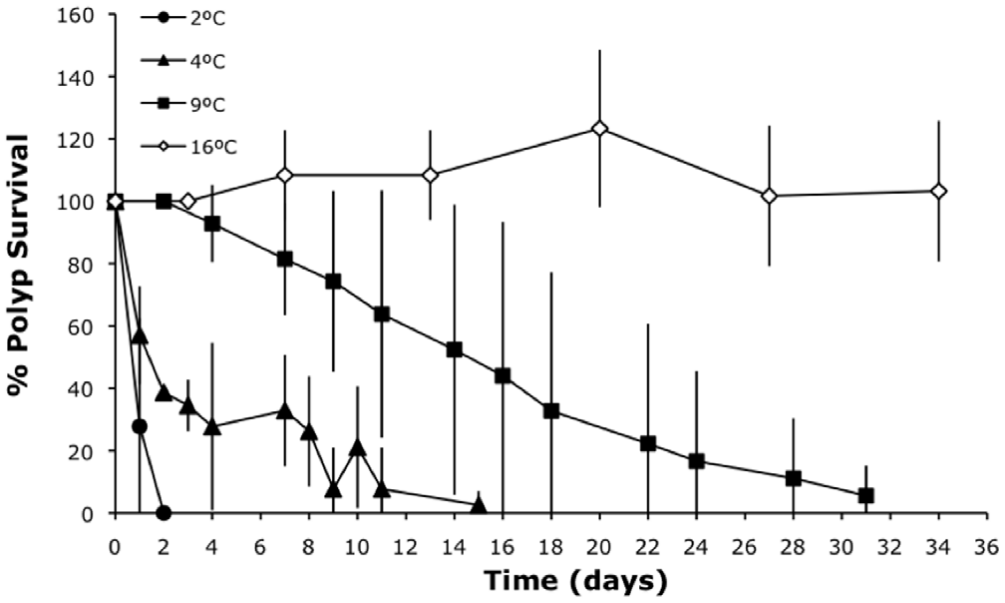
Polyp survival at low temperatures (Experiment E). Empty rhomboid, square, triangle and circle stand for 16, 9, 4 and 2°C respectively. Error bars are the standard deviation of the three replicates'. Survival is greater than 100% at 16°C because of budding.

Survival was very sensitive to temperatures below 16°C (Fig. 4). The time to reduce the total population to a half decreased from two weeks to one day for temperatures between 9 and 2°C, respectively (time range average: 15.7–1.3 days, F = 31.81, p<0.01).

### Transition from benthic to pelagic phase: strobilation

#### Experiment F: Strobilation at constant temperatures

**Figure 5:**
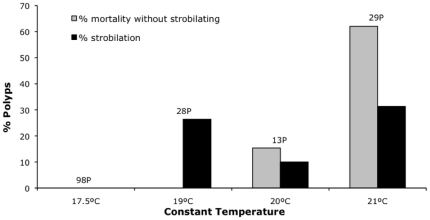
Strobilation at constant temperature (Experiment F). Previously, polyps were acclimated to a constant temperature (17.5, 19, 20 and 21°C) for 35 days without iodine in the water. Accumulate strobilation percentage of polyps and proportion of polyps that died without strobilating after 15 days of adding iodine, keeping the polyps at the same temperature at which they were acclimated. No polyps strobilated or died at 17.5°C. The total number of polyps at the beginning of the experiment is indicated.

Strobilation was inhibited below 19°C (Fig. 5), but 100% of polyps survived. As temperature increased polyps began to strobilate but the percentage of polyps that died without strobilating also increased (Fig. 5). To further understand the role of temperature change for strobilation, the flask of 17.5°C that showed no strobilation and no mortality after 15 days of adding iodine was moved to 20°C. 70% of the polyps strobilated after the temperature change, therefore resulting in higher strobilation than at a stable temperature of 20°C. To confirm this result, an experiment examining the effects of a change in temperature was performed (Experiment G).

#### Experiment G: Strobilation in incremental temperature increase

**Figure 6:**
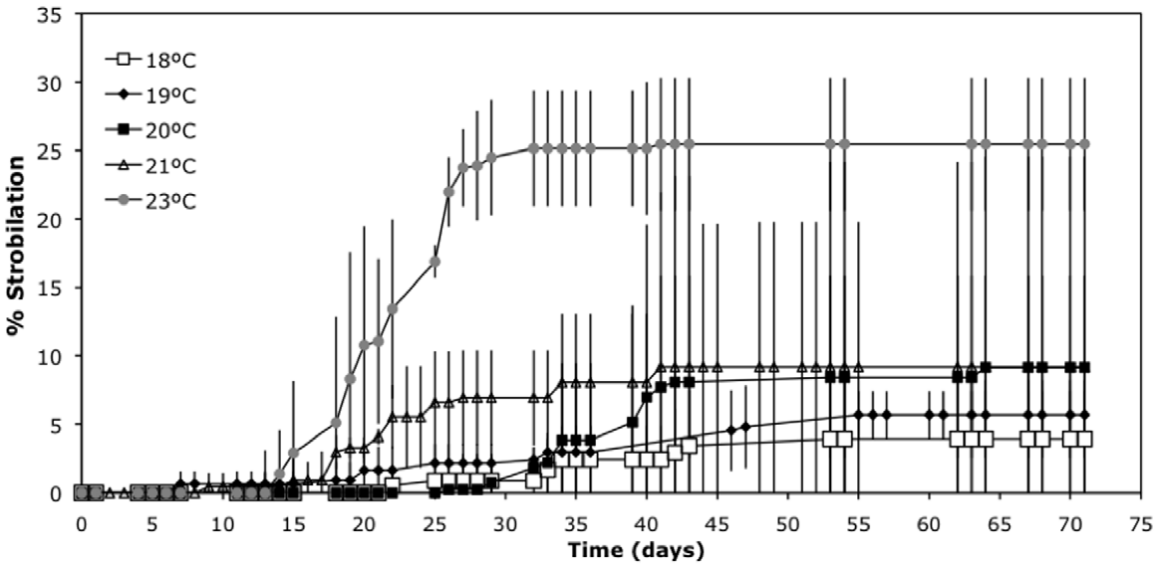
Triggering of strobilation by an increase in temperature (Experiment G). Cumulative percentage of polyp strobilation during a change of temperature from 17.5°C to 18, 19, 20, 21 and 23°C. Previously polyps were at 17.5°C for 5 months. The x-axis is the time (days) after the increment of temperature. Error bars are the standard deviation of the three replicates'.

The proportion of polyps that strobilated increased as the magnitude of the temperature change increased, i.e. maximum rates of strobilation occurred when the temperature was changed from 17.5 to 23°C (F = 15.93, p<0.01; Fig. 6).

On Day 40, when the maximum % of strobilation was reached, the proportion of polyps that died without strobilating was 16% (±21%) and 8% (±18%) for 21°C and 23°C, respectively, and zero for the remaining temperatures. All these polyps were kept unfed during the experiment, in contrast with the previous experiment at a stable temperature. As this no feeding treatment probably would have an effect on its own, the following experiment was performed (Experiment H).

#### Experiment H: Strobilation with increased temperature and different feeding conditions

**Figure 7:**
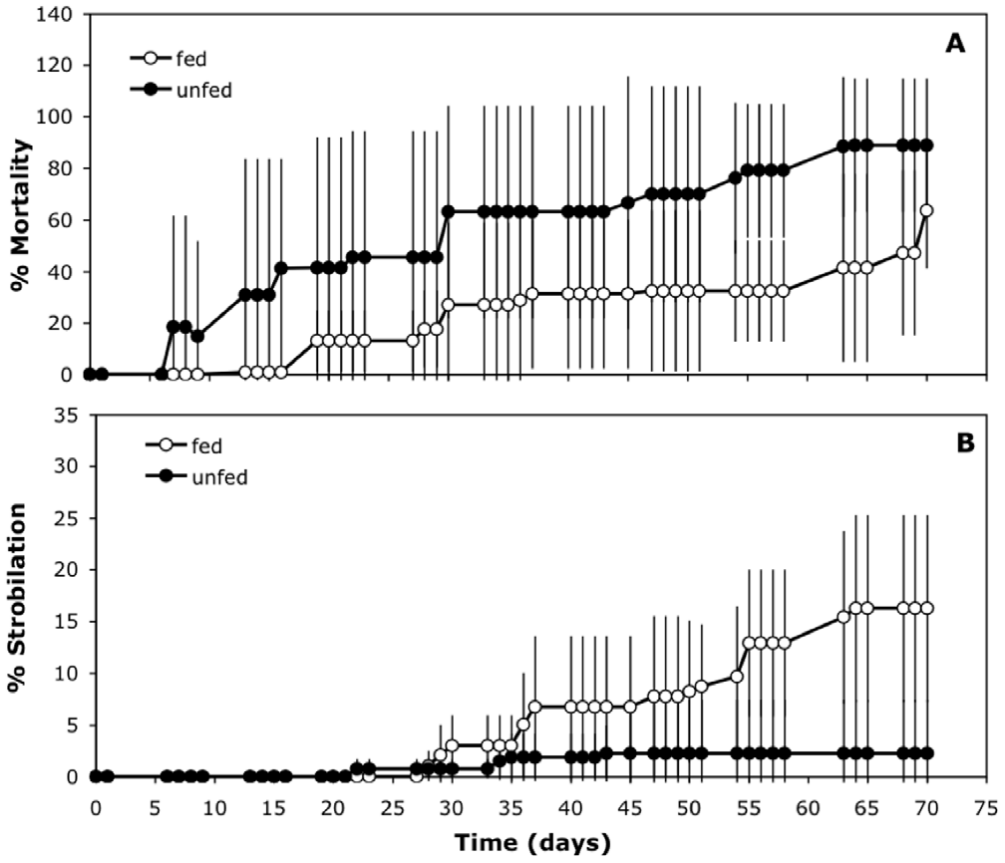
Effect of feeding on the percentage of strobilation during an increase in temperature (Experiment H). **A**) Proportion of polyps that died without strobilating during a change of temperature from 17.5°C to 26°C, fed with zooplankton (empty circles) and not fed (filled circles) during 70 days. **B**) Cumulative percentage of polyp strobilation in both treatments. Previously those polyps were kept at 17.5°C for 7 months. X-axis is the time (days) after the increment of temperature. Error bars are the standard deviation of the three replicates'.

Effectively, the proportion of polyps that died without strobilating increased in the unfed treatment with an increase of temperature to 26°C compared with the fed treatment, but the differences were not significant (F = 1.86, p>0.05; Fig. 7A). Meanwhile, the proportion of polyps that strobilated in the unfed treatment, reached its maximum after 35 days of the increase of temperature from 17.5°C to 26°C (Fig. 7B), as occurred in Experiment G. But in the treatment with zooplankton feeding, the proportion of polyps that strobilate kept increasing until day 65, when it reached a maximum of 16% (F = 8.15, p<0.05).

#### Experiment I: Strobilation with temperature fluctuations at synoptic time scales

Experiment I, which checked the drop of temperature from 20°C to 16°C for between 1 and 15 days, showed that no strobilation occurred in any of the treatments after more than a month back to 20°C.

**Figure 8:**
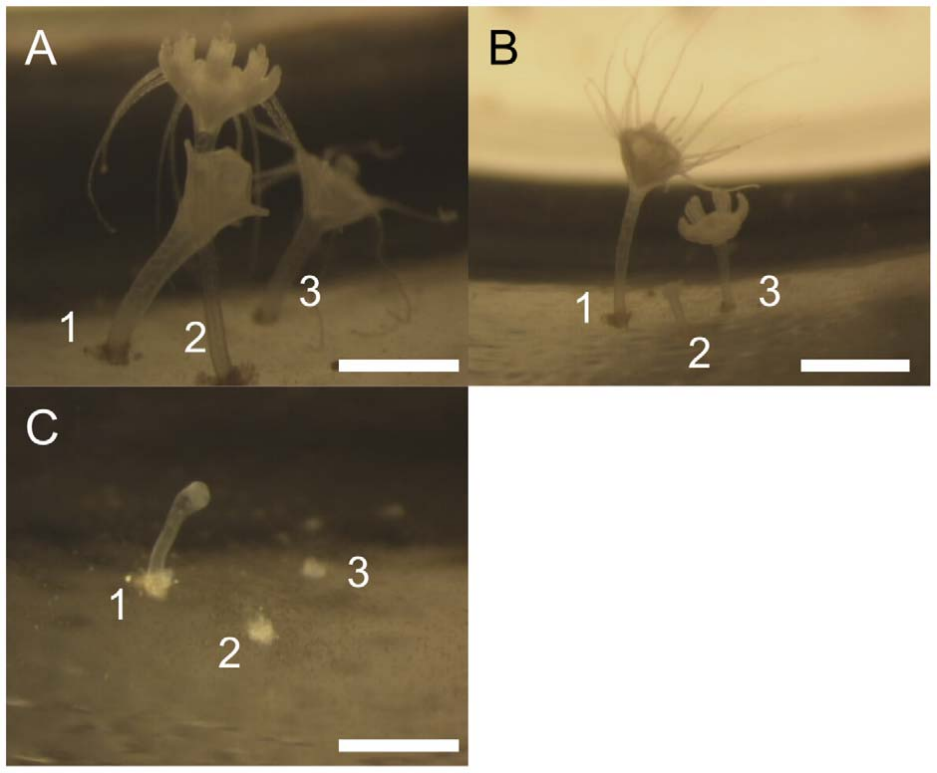
Developmental sequence of strobilating polyps. **A**) Polyp #1 after 1 day of strobilation, #2 in monodisc strobilation and #3 completely developed. **B**) Polyp #1 completely developed after 26 days of strobilation, #2 only stalk after liberating the ephyra and #3 in monodisc strobilation. **C**) Polyp #1 only stalk after liberating the second ephyra 40 days after liberating the first one, #2 and #3 died and only prints where pedal discs were attached are observed. All scale bars  = 500 µm. Author of the pictures: D. Astorga.

To fully understand this critical process in the *C. tuberculata* life cycle, a detailed monitoring of scyphistomae (observing them under the stereoscope everyday) was conducted. The time needed from the beginning of the metagenesis until the liberation of the ephyra was on average 14±3 days (n = 14). From a total of 10 flasks (with an average of 100 polyps in each one), 100% of mortality of the polyps was recorded in 7 flasks after the liberation of the ephyrae. From the remaining 3 flasks, 12 polyps that could be easily photographed under the steresocope, were selected and monitored every day for a total of 50 days to check the possibility of surviving after strobilation and repeated strobilation (Fig. 8). From the 12 polyps, 7 died after strobilation, 5 developed a complete scyphistoma with 16 tentacles after 25±8 days (n = 5), and only 2 could re-strobilated and liberated a second ephyra after 40 days of the first strobilation. Of the remaining 2 polyps, one died 9 days after the second strobilation, while the other was monitored for only two days after strobilation (survival is unknown). Thus, the mortality of polyps after strobilation was between 92±17% (n = 10, considering the flask as replicates) and 73% (considering the 11 polyps monitored after strobilation).

## Discussion

The life history of *Cotylorhiza tuberculata* in the Bay of Vlyho was described by Kikinger (1992). His detailed descriptions of the development of the natural population [28] are supplemented by observations of the present study, which reveal details of the control of the environment at different life stages, allowing its parameterization.

The sensitivity to temperature of planulae settlement and polyp development seems to be more related to the time needed to complete the process than the capacity to achieve it. Since the planula phase of the life cycle is highly resilient, temperature does not limit the generation of well developed polyps in late summer and early autumn. When temperatures are high enough, planulae become polyps within 2 to 4 weeks; lower temperatures simply extend this period. Thus, the planula is a phase that appears to be insensitive to the range of physical environment variables in the lagoon and is unlikely to be the critical step in the environmental control of the life cycle. Similarly, planulae (and polyps) from *Cyanea capillata* showed a high tolerance to low salinity [36], suggesting *C. tuberculata* planuale are able to persist until they find an adequate substrate where to attach and become a polyp.

Once polyps fix to the substrate during late summer and early autumn they must survive until the next spring when strobilation occurs. Although medusae are more visible, polyps are the main stage of *C. tuberculata* in the lagoon in terms of residence time. Pelagic stages of *C. tuberculata* happen from June to September whereas the benthic phase lasts for the rest of the year. Polyps must survive winter conditions when light and food are lower than in spring and summer. In addition, although polyp sensitivity to salinity was very low, temperature can drop dramatically in a shallow lagoon like Mar Menor. One example of this control of polyp survival by temperature occurred in 2005, when besides being one of the warmest months of June in 20 years (mean water temperatures of 23°C) no outbreak of *C. tuberculata* was detected within the lagoon [37]. To clarify this exception it is necessary to consider the effect of temperature not only on strobilation but also on polyp survival. Analyzing the air temperature and wind velocity at the meteorological station of the Agencia Estatal de Meteorología (AEMET) of San Javier airport (0.5 km from the inner shoreline of the lagoon), a strong cold event with temperatures close to freezing during several days in January 2005 was apparent. Such a severe and persistent cold event was not present from 1986 to 2005. In addition, this event was accompanied by strong winds (>20 km/h), which facilitate heat loss from this shallow lagoon in a period of very cold air temperature. Although there are no *in situ* data for the winter of 2005, the water temperature during these days must have dropped below 10°C and the polyps were exposed to severe stress (as evident from Fig. 4) probably resulting in a reduction of *C. tuberculata*'s polyp population in the lagoon. Thus, the high mortality of polyps at low winter temperatures seems to be a critical factor that controls polyp population in the Mar Menor. Natural populations of *Aurelia* sp. polyps monitored for two years in Tasmania waters also showed a significant relationship with mean water temperature [21]. Similarly, a temperature control was observed for the scyphomedusan *Rhopilema nomadica* in the Eastern Mediterranean [38] with temperatures below 13°C resulting in a high mortality of polyps.

A lack of light and/or nutrients did not affect polyp survival and the lack of feeding with rotifers could not be compensated by the photosynthetic activity of zooxanthellae. Consequently, zooxanthellae may play a modest role in the metabolism of polyps. Their presence neither prevented mortality during starvation nor increased survival when feeding with rotifers. Pitt et al. (2009) observed that *Cassiopea sp*. medusae when maintained unfed with light suitable for photosynthesis for their zooxanthellae, shrink, suggesting that zooplankton contain nutrients vital for regular metabolic function [39].

Budding seems to be stimulated by the absence of light in *C. tuberculata*. In other species such as *Aurelia labiata*, the intensity of low light (always below 10 µmol quanta m^−2^ s^−1^) had no effect on the number of buds per polyp, although it was different when the interaction between light and photoperiod were considered [40]. Experiments with light and temperature on tropical *Aurelia aurita* showed that the production of buds decreased with warmer temperature and stronger light intensity [41]. In winter, when the *C. tuberculata* polyps phase is present in the lagoon, light levels are lower, both in duration and intensity, probably favoring the budding process.

The success of asexual reproduction is dependent on both the strobilation process and the number of ephyrae released [42]. The high mortality of the population of *C. tuberculata* polyps after strobilation (up to 100%) makes it very unlikely that there is a second peak of ephyrae in the field, although there is a possibility that 12% of the population could perform a second liberation after 40 days. Comparing with other species of scyphozoans, *Rhopilema nomadica* [43], *Mastigias papua* [44] and *Stomolophus meleagris* [45] can liberate several ephyrae in the same season after multiple strobilations of the same polyp. Also, scyphozoans such as *Lychnorhiza lucerna* [46], *Nemopilema nomurai* [47] or *Rhizostoma pulmo* [48] are polydisk and therefore can produce up to 17 ephyrae per strobila. As the percentage of polyps of *C. tuberculata* that died without strobilation increased at higher temperatures, even when high food supply occurred (Fig. 7), the only way for the population to survive to the summer is by means of the medusae stage. Lotan et al. (1994) also found a decline in strobilation of *Rhopilema nomadica* occurred at high temperatures (24 and 26°C [38]) and many polyps of *Aurelia labiata* died without strobilating at 30°C [41]. Consequently, *C. tuberculata* may be considered a jellyfish with an annual life-span, since a very high proportion of the polyps dies after a monodisc-type strobilation has occurred and the remaining do not survive well in warm temperatures (typical of the spring/summer season). This finding is also corroborated by the *C. tuberculata* population in the Bay of Vhylo, with a one year life-span, suggesting this maybe a general characteristic of the species [28].

As an increase in temperature results in higher strobilation rates than at stable temperature, and to parametrize this year life span cycle, Experiment I checked the seasonal against the synoptic effect (meteorological event with a time scale between days to weeks) of the environment temperature to trigger the strobilation process. No strobilation occurred in any of the treatments, confirming that the increase in temperature required to trigger strobilation in *C. tuberculata* needs to be at a seasonal scale, and a synoptic event (i.e. a storm, a drop of temperatures associated to a low pressure situation) is not sufficient. Again this fits well with data on the *C. tuberculata* population of the Bay of Vhylo, which was found to have a peak of ephyrae release at seasonal time scale (mid June) representing an increase in temperature from 20°C to 24°C [28].

In summary, this article provides a reliable, robust example of how temperature controls the population of a massive-outbreaker jellyfish. Early life stages of *Cotylorhiza tuberculata* are not sensitive to salinity variations or the availability of light or nutrients. However, temperature critically controls polyp survival and strobilation. Low temperatures imply reduced polyp survival during the winter. Abrupt water warming during spring triggers strobilation and, therefore, the start of the medusa phase of the life cycle. In coherence with laboratory results, these thermal controls determine the inter-annual presence/absence of outbreaks of this jellyfish in the Mar Menor lagoon. Projecting this thermal control on the life cycle of *Cotylorhiza tuberculata* within the framework of future climate scenarios [49], i.e. milder winters and hotter summers, suggests blooms of this jellyfish will be increasingly recurrent in the Mediterranean Sea.

## References

[1] Mills CE (2001). Jellyfish blooms: Are populations increasing globally in response to changing ocean conditions?. Hydrobiologia.

[2] Shiganova TA, Bulgakova YV, Musaeva EI, Mirzoyan ZA, Martynyuk ML (2003). Invaders Ctenophores *Mnemiopsis leidyi* (A. Agassiz) and *Beroe ovata* Mayer 1912, and Their Influence on the Pelagic Ecosystem of Northeastern Black Sea. Bull.

[3] Malej A, Turk V, Lucic D, Benovic A (2007). Direct and indirect trophic interactions of *Aurelia* sp. (Scyphozoa) in a stratified marine environment (Mljet Lakes, Adriatic Sea).. Mar Biol.

[4] Purcell JE, Uye SI, Lo WT (2007). Anthropogenic causes of jellyfish blooms and direct consequences for humans: a review.. Mar Ecol Prog Ser.

[5] Yamamoto J, Hirose M, Ohtami T, Sugimoto K, Hirase K (2008). Transportation of organic matter to the sea floor by carrion falls of the giant jellyfish *Nemopilema nomurai* in the Sea of Japan.. Mar Biol.

[6] Pitt KA, Purcell JE (2009). Jellyfish blooms: Causes, consequences, and recent advances.. Hydrobiologia.

[7] Purcell JE, Angel D (2010). Jellyfish blooms: New problems and solutions.. Hydrobiologia.

[8] Arai MN (2001). Pelagic coelenterates and eutrophication: a review.. Hydrobiologia.

[9] Parsons TR, Lalli CM (2002). Jellyfish population explosions: Revisiting a hypothesis of possible causes.. La Mer.

[10] Holst S, Jarms G (2007). Substrate choice and settlement preferences of planula larvae of five Scyphozoa (Cnidaria) from German Bight, North Sea.. Mar Biol.

[11] Purcell JE, Shiganova TA, Decker MB, Houde ED (2001). The ctenophore *Mnemiopsis* in native and exotic habitats: US estuaries versus the Black Sea basin.. Hydrobiologia.

[12] Pauly D, Christensen V, Guénette S, Pitcher TJ, Sumaila UR (2002). Towards sustainability in world fisheries.. Nature.

[13] Purcell JE (2005). Climate effects on formation of jellyfish and ctenophore blooms.. J Mar Biol Assoc U K.

[14] Gibbons MJ, Richardson AJ (2009). Patterns of jellyfish abundance in the North Atlantic.. Hydrobiologia.

[15] Sugiura Y (1965). On the life-history of rhizostome medusae. III. On the effect of temperature on the strobilation of *Mastigias papua*.. Biol Bull.

[16] Calder DR (1973). Laboratory observations on the life history of *Rhopilema verrilli* (Scyphozoa: Rhizostomae).. Mar Biol.

[17] Hofmann DK, Neumann R, Henne K (1978). Strobilation, budding and initiation of scyphistoma morphogenesis in the rhizostome *Cassiopea andromeda*.. Mar Biol.

[18] Lucas CH (2001). Reproduction and life history strategies of the common jellyfish, *Aurelia aurita*, in relation to its ambient environment.. Hydrobiologia.

[19] Colin SP, Kremer P (2002). Population maintenance of the scyphozoan *Cyanea* sp. settled planulae and the distribution of medusae in the Niantic River, Connecticut, USA.. Estuaries.

[20] Barz K, Hirche H-J (2007). Abundance, distribution and prey composition of scyphomedusae in the southern North Sea.. Mar Biol.

[21] Willcox S, Moltschaniwskyj NA, Crawford CM (2008). Population dynamics of natural colonies of *Aurelia* sp. scyphistomae in Tasmania, Australia.. Mar Biol.

[22] Hoover RA, Purcell JE (2009). Substrate preferences of scyphozoan *Aurelia labiata* polyps among common dock-building materials.. Hydrobiologia.

[23] Shiganova TA, Mirzoyan ZA, Studenikina EA, Volovik SP, Siokou-Frangou I (2001). Population development of the invader ctenophore, *Mnemiopsis leidyi*, in the Black Sea and in other seas of the Mediterranean basin.. Mar Biol.

[24] Malej A, Malej A, Dumont H, Shiganova TA, Niermann U (2004). Invasion of the jellyfish *Pelagia noctiluca* in the Northern Adriatic: a non-success story.. Aquatic invasions in the Black, Caspian and Mediterranean Seas.

[25] Kogovsek T, Bogunovic B, Malej A (2010). Recurrence of bloom-forming scyphomedusae: wavelet analysis of a 200-year time series.. Hydrobiologia.

[26] E C O S (2004). Poblaciones de medusas en el Mar Menor.. Murcia: C A R M.

[27] Perez-Ruzafa A, Gilabert J, Gutiérrez JM, Fernández AI, Marcos C (2002). Evidence of a planktonic food web response to changes in nutrient input dynamics in the Mar Menor coastal lagoon, Spain.. Hydrobiologia.

[28] Kikinger R (1992). *Cotylorhiza tuberculata* (Cnidaria: Scyphozoa) life history of a stationary population.. P S Z N I Mar Ecol.

[29] Pagès F, Briand F (2001). Past and present anthropogenic factors promoting the invasion, colonization and dominance by jellyfish of a Spanish coastal lagoon.. Gelatinous zooplankton outbreaks: theory and practice.

[30] Santos SR, Gulko D, Jokiel PL (1995). Ultraviolet radiation: helpful or harmful to growth of cultured zooxanthellae?. Ultraviolet Radiation and Coral Reefs. H I M B Tech Report 41.

[31] Velasco J, Lloret J, Millán A, Marín A, Barahona J (2006). Nutrient and particulate inputs into the Mar Menor lagoon (SE Spain) from an intensive agricultural watershed.. Water Air Soil Pollut.

[32] Spangenberg DB (1965). A study of strobilation in *Aurelia aurita* under controlled conditions.. J Exp Zool.

[33] Silverstone M, Tostenson TR, Cutress CE (1977). The effect of iodine and various iodocompounds on initiation of strobilation in *Aurelia*.. Gen Comp Endocrinol.

[34] Berking S, Czezh N, Gerharz M, Herrmann K, Hoffmann U (2005). A newly discovered oxydant defence system and its involvement in the development of *Aurelia aurita* (Scyphozoa, Cnidaria): reactive oxygen species and elemental iodine control medusa formation.. Int J Dev Biol.

[35] Pierce J (2005). A system for mass culture of upside-down jellyfish as a potential food item for medusivores in captivity.. Int Zoo Yb.

[36] Holst S, Jarms G (2010). Effects of low salinity on settlement and strobilation of Scyphozoa (Cnidaria): Is the lion's mane *Cyanea capillata* (L.) able to reproduce in the brackish Baltic Sea?. Hydrobiologia.

[37] Pellicier M (2007). http://www.20minutos.es/noticia/248656/0/verano/finales/junio/.

[38] Lotan A, Fine M, Ben-Hillel R (1994). Synchronization of the life cycle and dispersal pattern of the tropical invader scyphomedusan *Rhopilema nomadica* is temperature dependent.. Mar Ecol Prog Ser.

[39] Pitt KA, Welsh DT, Condon RH (2009). Influence of jellyfish blooms on carbon, nitrogen and phosphorus cycling and plankton production.. Hydrobiologia.

[40] Purcell JE (2007). Environmental effects on asexual reproduction rates on the scyphozoan *Aurelia labiata*.. Mar Ecol Prog Ser.

[41] Liu WC, Lo WT, Purcell JE, Chang HS (2009). Effects of temperature and light intensity on asexual reproduction of the scyphozoan *Aurelia aurita* (L.) in Taiwan.. Hidrobiologia.

[42] Henroth L, Grondahl F (1983). On the release of *Aurelia aurita* (L.) 1 Release and growth of *Aurelia aurita* (L.) ephyrae in the Gullmar fjord, western Sweden, 1982–83.. Ophelia.

[43] Lotan A, Ben-Hillel R, Loya Y (1992). Life cycle of *Rhopilema nomadica*: a new immigrant scyphomedusan in the Mediterranean.. Mar Biol (Berl).

[44] Sugiura Y (1963). On the life history of rhizostome medusa I. *Mastigias papua* L. Agassiz.. Annot Zool Jpn.

[45] Calder DR (1982). Life history of the Cannonball jellyfish, *Stomolophus meleagris* L. Agassiz, 1860 (Scyphozoa, Rhizostomida).. Biol Bull.

[46] Schiriati A, Kawahara M, Uye S, Mianzan HW (2008). Life cycle of the jellyfish *Lychnorhiza lucerna* (Scyphozoa: Rhizostomeae).. Mar Biol.

[47] Kawahara M, Uye SI, Ohtsu K, Iizumi H (2006). Unusual population explosion of the giant jellyfish *Nemopilema nomurai* (Scyphozoa: Rhizostomeae) in East Asian waters.. Mar Ecol Prog Ser.

[48] Paspaleff BW (1938). Über die Entwicklung von *Rhizostoma pulmo* Agassiz.. Trud chernomorsk biol Sta Varna.

[49] Hansen J, Sato M, Ruedy R, Lo K, Lea DV (2006). Global temperature change.. Proc Natl Acad Sci U S A.

